# Venoarterial Extracorporeal Membrane Oxygenation-Assisted Whole-Lung Lavage for Near Drowning With Massive Sand Aspiration: A Case Report

**DOI:** 10.7759/cureus.94320

**Published:** 2025-10-10

**Authors:** Tsuyoshi Suzuki, Chiaki Nemoto, Reiko Okubo, Makoto Onodera, Ken Iseki

**Affiliations:** 1 Department of Emergency and Critical Care Medicine, Fukushima Medical University, Fukushima, JPN; 2 Department of Regional Emergency Medicine, Fukushima Medical University, Fukushima, JPN

**Keywords:** bronchoalveolar lavage, drowning, extra-corporeal membrane oxygenation, prone position, whole-lung lavage

## Abstract

Sand aspiration during drowning can cause tracheobronchial obstruction and severe respiratory failure. In life-threatening hypoxemia, there is currently no established method for the safe removal of aspirated sand. We report a case of a 50-year-old man with hypertension and angina pectoris who nearly drowned while surfing. Upon arrival at the hospital, he was unconscious, hypoxemic, and hypothermic. Chest computed tomography revealed extensive bilateral dorsal infiltrates and high-density material within the bronchi. Despite repeated suctioning of the foul-smelling sand, the partial pressure of arterial oxygen (PaO_2_)/fraction of inspired oxygen (FiO₂) (P/F) ratio remained less than 100 even with an FiO₂ of 1.0, necessitating whole-lung lavage (WLL). Transthoracic echocardiography (TTE) revealed severely impaired cardiac function. Because of the risk of cardiac arrest, WLL was initiated under venoarterial extracorporeal membrane oxygenation (VA-ECMO). Shortly after WLL began, the patient developed bradycardia and cardiac arrest but returned to spontaneous circulation within minutes. WLL successfully cleared the aspirated sand. His pneumonia improved, and he regained the ability to communicate. On day 49 of hospitalization, the patient was transferred for rehabilitation. Although extensive WLL is generally avoided in sand aspiration, it may be warranted in cases of severe hypoxemia caused by widespread particulate aspiration. In patients with impaired cardiac function, performing WLL under VA-ECMO support may represent a safer approach.

## Introduction

In outdoor drowning, not only microorganisms but also particulate matter, such as sand and mud, enter the alveoli, leading to airway obstruction. Drowning is a known risk factor for drowning-associated pneumonia (DAP) and acute respiratory distress syndrome. Removing particulate matter from the alveoli and airways is essential to achieve resolution of respiratory failure. Whole-lung lavage (WLL) has been established as an effective method for washing the trachea during pulmonary alveolar proteinosis [1,2]. However, previously, there have been reports of WLL in adults due to aspiration of lubricants [3]; no report has described the use of WLL for near drowning with sand aspiration. Moreover, the indications for WLL in drowning-related, life-threatening respiratory failure due to sand aspiration remain unclear. Here, we describe the successful use of venoarterial extracorporeal membrane oxygenation (VA-ECMO)-assisted WLL in a patient with severe hypoxemia following drowning and massive sand aspiration.

## Case presentation

**Figure 1:**
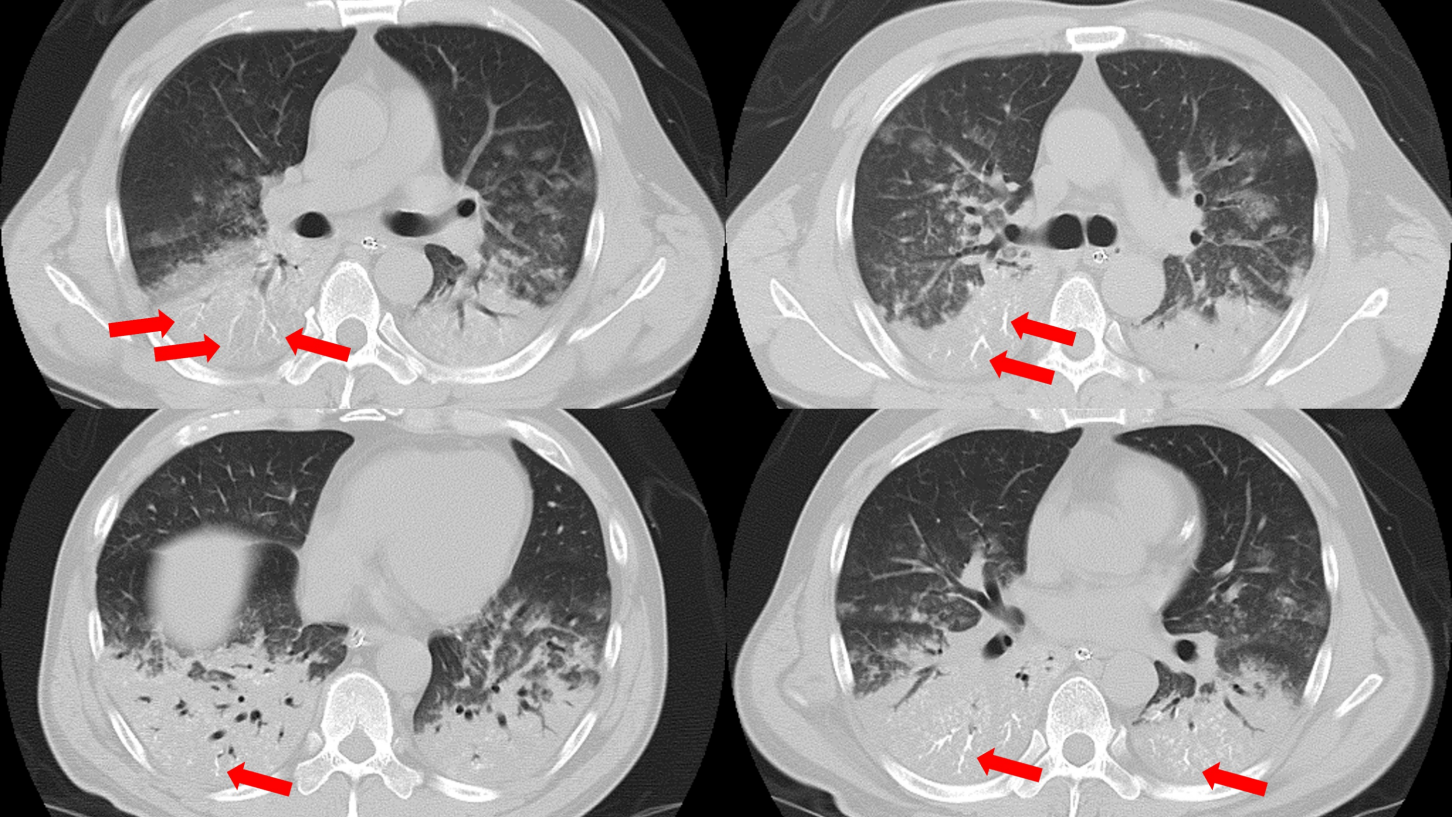
Axial chest computed tomography image on admission Extensive infiltrative shadows were observed in the bilateral dorsal lungs. In addition, there was a high-density finding of gravel and mud in the bronchi of the same area (red arrow).

A 50-year-old man with a history of hypertension, angina pectoris, and smoking (20 cigarettes/day) disappeared while surfing. Approximately one hour later, the patient was found unconscious and transferred to a hospital. On arrival, his Glasgow Coma Scale (GCS) score was three, respiratory rate 30 breaths/min, oxygen saturation (SpO_2_) 75% (room air), blood pressure 213/146 mmHg, heart rate 94 beats per minute (bpm), and body temperature 33°C. He had impaired consciousness and hypoxemia and was intubated. During intubation, large amounts of sand and mud were aspirated from the trachea, which were thought to be due to drowning (Figure 1).

After intubation, his SpO_2_ remained low at 80% despite a fraction of inspired oxygen (FiO_2_) of 1.0. The patient was transferred to our hospital for intensive care. He remained unconscious and hypoxemic. Blood tests (Table 1) showed a white blood cell count of 2400/µL, hemoglobin 17.2 g/dL, platelet count 33.4×104/µL/µL, prothrombin time/international normalized ratio 1.0, activated partial thromboplastin time 24.4 sec, glutamic oxaloacetic transaminase 44 U/L, glutamic pyruvic transaminase 57 U/L, lactate dehydrogenase 238 U/L, creatinine 1.0 mg/dL, Na^+^ 155 mEq/L, K^+^ 4.2 mEq/L, and Cl 125 mEq/L. Blood gas analysis (FiO_2_ 1.0) showed pH 7.144, partial pressure of arterial oxygen (PaO_2_) 54.2 mmHg, partial pressure of arterial carbon dioxide (PaCO_2_) 56.7 mmHg, and bicarbonate (HCO3) 18.7 mmol/L. 

**Table 1:** Blood exams on admission. WBC: white blood count; PLT: platelet; PT: prothrombin time; APTT: activated partial thromboplastin time; AST: aspartate aminotransferase; ALT: alanine transaminase; BNP: brain natriuretic peptide; CRP: C-reactive protein

Laboratory parameters	Value	Reference range
WBC	2.4	3.8-9.8(x10^3^/μL)
Hemoglobin	17.2	13.2-16.8(g/dL)
PLT	33.6	14.7-34.1(x10^4^/μL)
PT	90.2	70-125(%)
APTT	24.4	23-38(sec)
Total protein	7.6	6.7-8.3(g/dL)
Serum albumin	4.3	3.9-4.9(g/dL)
Creatinine	1	0.6-1.1(mg/dL)
AST	44	10-38(U/L)
ALT	57	8-42(U/L)
Total bilirubin	0.5	0.2-1.2mg/dL)
Sodium	155	138-146(mEq/L)
Potassium	4.2	3.6-4.9(mEq/L)
Chloride	125	99-106(mEq/L)
Troponin I	<0.017	0.00-0.056(ng/mL)
BNP	14.3	0.0-18.4(pg/mL)
CRP	7.04	<0.03(mg/dL)

**Figure 2:**
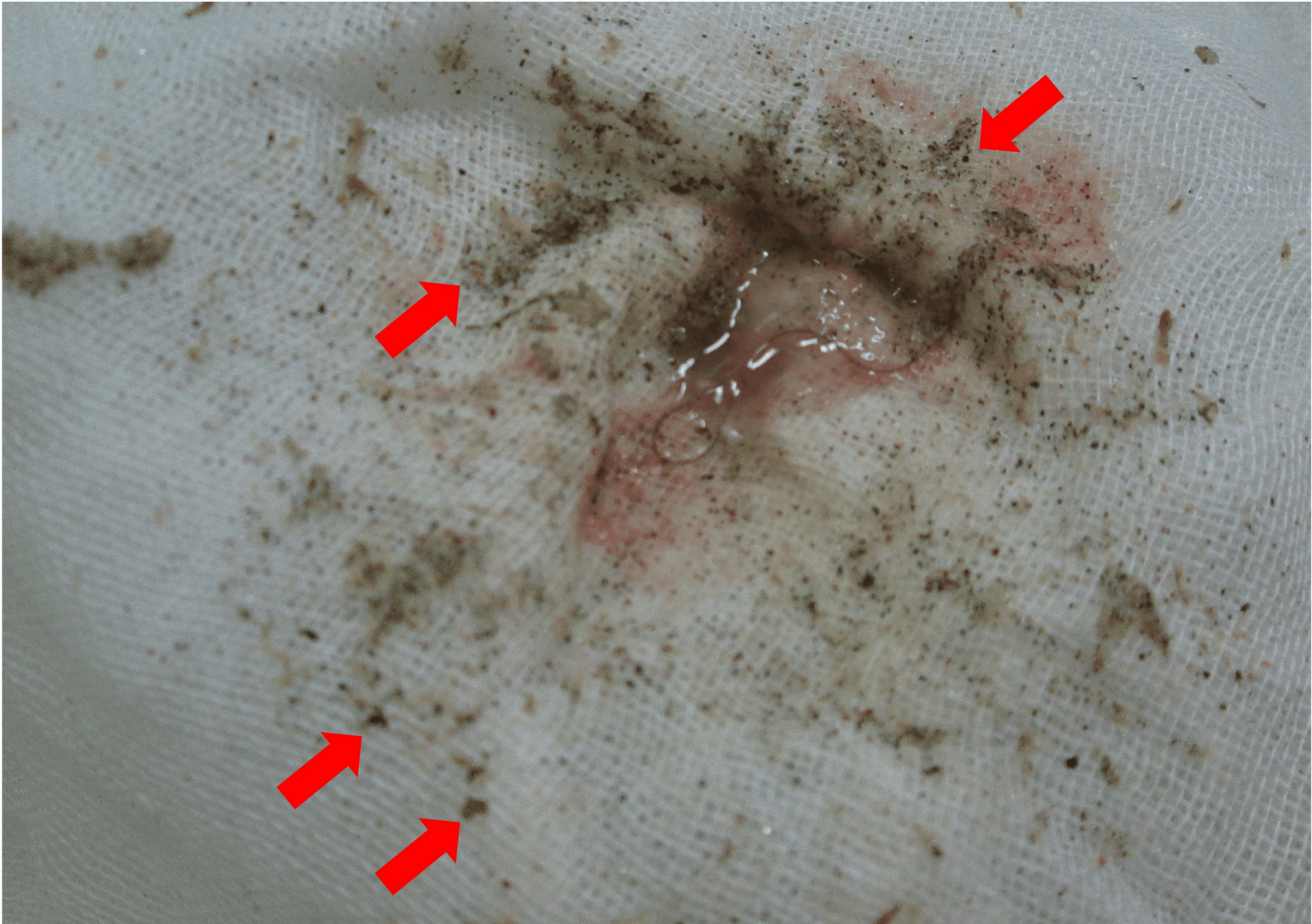
Small particles obtained via the whole lung lavage (WLL) The washing solution obtained during the first WLL cycle, filtered through gauze. Countless small dots, likely sand particles (red arrows), are observed.

Transthoracic echocardiography (TTE) revealed a left ventricular ejection fraction of 25%-30%; diffuse left ventricular hypokinesis was shown. After admission to the intensive care unit, a large amount of sand was aspirated from the trachea. Therefore, the patient was diagnosed with near drowning with aspirated sand and mud. The presence of hypernatremia also suggested drowning in seawater. Meropenem (1 g every 12 hours) was administered as antibiotic therapy. Invasive positive-pressure ventilation was then initiated (assist-control mode; FiO_2_, 1.0; respiratory rate, 12/min; positive end-expiratory pressure (PEEP), 10 cmH_2_O); however, to achieve a tidal volume of 6 mL・kg⁻¹ of predicted body weight, the peak airway pressure (PAP) reached 40 cmH_2_O, and the PaO_2_/FiO_2_ ratio was <100. Despite several attempts at endotracheal suction over one to two hours, the patient showed no change in PAP and hypoxemia. Thus, endotracheal suctioning was insufficient for sand removal, and WLL was planned. To prepare for WLL, the tracheal tube was changed to a double-lumen tube. Isolated lung ventilation and WLL were planned for the non-ventilated side. However, severe hypoxemia reduced cardiac function on TTE, and worsening hypoxemia during WLL raised concerns about cardiac arrest. Therefore, we decided to initiate VA-ECMO and then perform WLL to remove the aspirated particles. On day 1 of admission, VA-ECMO (MERA centrifugal blood pump system HCS-CFP; Senko Medical Instruments, Tokyo, Japan) was initiated. We inserted a 20 Fr cannula (PCKC-A; MERA Development Corp., Tokyo, Japan) into the right femoral artery and a 24 Fr cannula (PCKC-V; MERA Development Corp) into the right femoral vein and established VA-ECMO (3000 rpm; pump flow 3.0 L/min; O₂ flow 2.0 L/min). WLL was initiated with warm normal saline, and the patient soon experienced cardiac arrest following severe bradycardia. The ECMO flow was increased to 5.1 L/min, followed by resuscitation for a few minutes until return of spontaneous circulation was achieved. We continued the WLL and obtained alveolar lavage fluid that appeared muddy. It smelled foul, and when we filtered it through gauze, we observed numerous small, sand-like particles (Figure 2).

**Figure 3:**
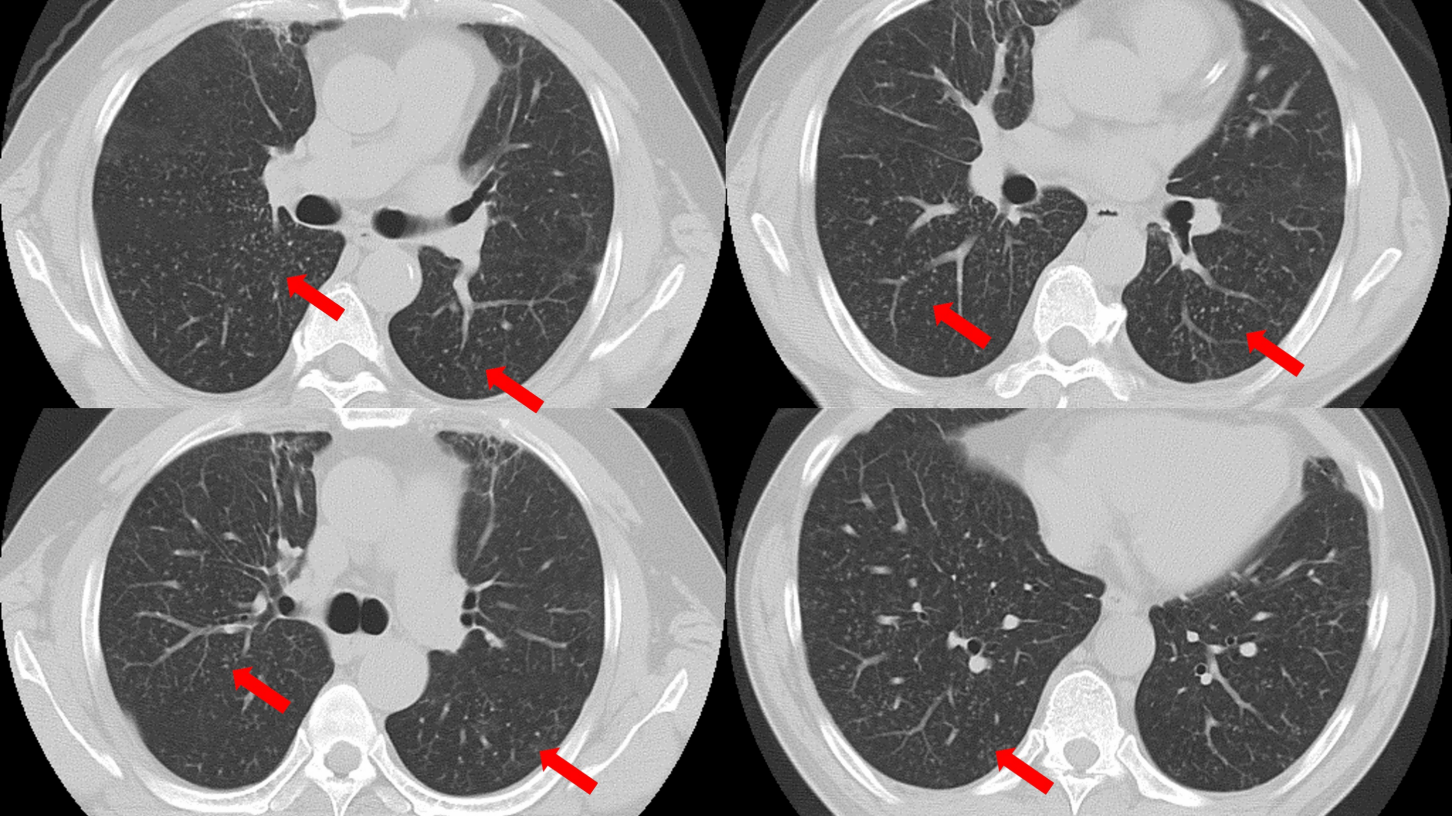
Axial chest computed tomography image one year later The diffuse infiltrative shadow had disappeared, leaving only a slight miliary shadow along the microbronchi.

The treatment was stopped when the wash solution became clear (5 liters × 5 cycles, total 25 L). Following WLL, PAP decreased to 30 cmH₂O. On day three, cardiac function improved, but severe respiratory failure persisted; therefore, a 24 Fr blood cannula (PCKC-A; MERA Development Corp) was inserted into the right internal jugular vein, and the circuit was converted from VA-ECMO to venovenous (VV) ECMO. On day seven, the patient was weaned off VV-ECMO; however, on day nine, VV-ECMO was reintroduced because of hypercapnia with decreased pulmonary compliance. On day 18, a tracheostomy was performed. On day 30, the patient was weaned off the ventilator. On day 49, he was transferred to the rehabilitation department because of muscle weakness. He fully recovered after hospitalization one year later. Respiratory function tests showed that his vital capacity recovered to 81%, and his forced expiratory volume in one second was three liters. Computed tomography revealed the disappearance of diffuse infiltrative shadows in the lungs, with only a few miliary shadows remaining along the bronchioles (Figure 3).

## Discussion

This case demonstrates that WLL under VA-ECMO support can save the lives of patients with severe hypoxemia caused by extensive sand aspiration. In cases of severe hypoxemia with shock, WLL with VA-ECMO may minimize the risk of worsening hypoxemia and cardiovascular instability. No cases of WLL for near drowning with sand aspiration have been reported, and evidence for its safety is lacking. WLL is usually indicated for alveolar proteinosis and is considered an overall safe procedure [1], although complications such as hypoxemia, pleural effusion, and pneumothorax have been reported [4]. Centella et al. reported a case of cardiac arrest after WLL in a young man with alveolar proteinosis and severe hypoxemia [5]. WLL with VV-ECMO was performed after resuscitation. The patient in that case was hypoxemic despite ventilatory management on admission (assist-control mode; pressure control 12 cmH₂O; PEEP 10 cmH₂O; FiO₂ 1.0), with a systolic blood pressure of 80 mmHg and a heart rate of 150 bpm, and was in shock; therefore, WLL was considered difficult to perform. In contrast, sand and mud cause extensive peripheral airway obstruction, resulting in increased airway pressure and severe hypoxemia, making early removal of the aspirated material necessary. In this case, although PAP decreased after the WLL, improvement in oxygenation was insufficient, necessitating a switch to VV-ECMO. While the WLL may have prevented secondary pulmonary barotrauma, it is unclear whether it contributed to reducing pulmonary inflammation.

We performed WLL with VA-ECMO support. VV-ECMO may only be considered for severe hypoxemia without shock. VA-ECMO with preserved left ventricular function is associated with increased cardiac afterload. Once cardiac function improves, switching from VA-ECMO to VV-ECMO, as in this case, may also be beneficial. This procedure should not be applied to all drowning patients who have aspirated sand; its applicability must be carefully considered. It should be carefully evaluated in cases with impaired cardiac function. 

WLL and bronchoalveolar lavage (BAL) may also be useful for removing small aspirated materials from the alveoli. Ramirez et al. [6] reported the use of WLL to treat alveolar proteinosis. Recently, Shang et al. [7] reported its use in treating exogenous lipoid pneumonia. In WLL, isolated lung ventilation is performed under general anesthesia, and the non-ventilated lung is washed with saline solution. For alveolar proteinosis, lung lavage is performed until 5-40 liters of saline become clear [8]. In this case, WLL was repeated five times, each time using 5 liters of 37℃ normal saline. However, WLL is not widely used to remove aspirated material in sand aspiration and should be considered only when aspirated material is widely distributed throughout the lung field, endotracheal suctioning is inadequate to remove aspirated material, and hypoxemia is present. In addition, BAL, which is less invasive than WLL, should be considered. In 2009, Kapur et al. [9] performed BAL to remove aspirated sand and administered surfactant. BAL may be more effective than WLL when the aspirated material does not extend to the whole lung field but remains at a certain segmental branch level.

During the WLL procedure, the patient was placed in the prone position. Although several positions for patients undergoing WLL have been reported, the optimal position remains unknown. In alveolar proteinosis, the supine position, the lateral position with the washed lung above, the lateral position with the washed lung below, and the prone position have been reported [2,10]. The aspirated material usually accumulates dorsally rather than ventrally. Because sand has a higher specific gravity than water, the prone position may facilitate dorsal drainage during WLL with sand suction after drowning. In our case, WLL was initially performed in the supine position, but only a small amount of sand was discharged into the wash solution. The patient was then repositioned to the prone position, and sand discharge was successfully achieved. However, careful attention should be paid to circulatory changes associated with changes in position because complications such as hypotension and hypoxemia may occur in the prone position. In this case, the patient was placed in the prone position during VA-ECMO. Although hypotension and hypoxemia developed, the procedure was performed safely with precautions specific to patients on ECMO in the prone position, such as preventing the cannula from kinking and ensuring proper blood suctioning of the ECMO circuit.

## Conclusions

Herein, we present a rare case of drowning-related massive sand aspiration successfully managed using VA-ECMO-assisted WLL. This approach may be considered for patients with severe hypoxemia and impaired cardiac function when conventional suction fails. Further studies are required to clarify the safety and efficacy of this strategy.
